# The Correlation of Clinical, Lipid- and Inflammation-Based Indices with Gensini Score in Young NSTEMI Patients

**DOI:** 10.3390/jcm15197442

**Published:** 2026-09-24

**Authors:** Şükriye Uslu, Gülsüm Meral Yılmaz Öztekin, Muhammed Rıdvan Ersoysal, Sadık Barış Adıgüzel, Sevi Karakaya, Görkem Kuş, Oğuz Kaan Kaya, Şakir Arslan

**Affiliations:** 1 Department of Cardiology, Antalya Training and Research Hospital, Health Science University, Antalya 07100, Turkey; 2 Department of Family Medicine, Antalya Training and Research Hospital, Health Science University, Antalya 07100, Turkey

**Keywords:** premature myocardial infarction, young NSTEMI patients, Gensini score, coronary atherosclerosis, smoking, Castelli risk index 2

## Abstract

**Background/Objectives:** Lipid-derived and lymphocyte-based inflammatory indices (LBIIs) are studied in coronary artery disease (CAD), but their relationship with angiographic burden in patients aged ≤45 years with non-ST-elevation myocardial infarction (NSTEMI) remains unclear. We evaluated clinical, laboratory, lipid-derived, and LBII correlates of the Gensini score. **Methods:** In this retrospective cross-sectional study, 402 troponin-positive NSTEMI patients undergoing coronary angiography were screened; 288 of them were eligible. Univariable associations were assessed using Spearman correlations with Benjamini–Hochberg false-discovery-rate (FDR) correction. Multivariable linear regression of log2 (Gensini + 1), with HC3-robust standard errors, included 281 complete cases. The selection of CRI-2 after inspection of the univariable results was exploratory rather than prespecified. Raw, square-root, and median models assessed sensitivity. **Results:** Mean age was 40.7 ± 4.1 years; 83.3% of patients were male and 76.0% were current smokers, and median Gensini score was 32.0 (IQR 9.4–58.1). Multivariable analysis showed greater burden with current smoking (+114.3%, *p* < 0.001), male sex (+90.1%, *p* = 0.013), each 5-year age increase (+33.9%, *p* = 0.009), and each 1-unit Castelli risk index-2 (CRI-2) increase (+23.3%, *p* = 0.009); a 5-percentage-point left ventricular ejection fraction (LVEF) increase was associated with an 18.4% lower burden (*p* = 0.001). None of the five LBIIs showed a statistically significant association with the continuous Gensini score after Benjamini–Hochberg FDR correction in univariable screening. Smoking, sex, age, and LVEF remained consistent across scales, whereas CRI-2 was statistically significant in the log2-transformed and square-root-transformed models but not in the raw and median models. **Conclusions:** In young patients with NSTEMI, smoking, male sex, older age, and higher CRI-2 were associated with greater coronary atherosclerotic burden, whereas none of the examined LBIIs showed a statistically significant association with coronary atherosclerotic burden after FDR correction. These findings reinforce the importance of smoking cessation and guideline-directed lipid management in secondary prevention.

## 1. Introduction

Coronary artery disease (CAD) and acute myocardial infarction (MI) are among the leading causes of morbidity and mortality worldwide [1]. Although CAD has traditionally been observed in older individuals, there has been a marked increase in the incidence of premature MI in recent years [2,3]. Premature CAD is a chronic and aggressive disease characterized by a high rate of recurrence and rapid progression to multivessel disease [2,3]. The observed increase in the incidence of non-ST-elevation myocardial infarction (NSTEMI) among young adults indicates that premature MI constitutes a substantial clinical burden and an important cause of morbidity and mortality that cannot be overlooked [4]. The higher frequency of multivessel disease among young patients with NSTEMI than among young patients with STEMI suggests that the coronary atherosclerotic burden underlying premature MI may be more extensive and advanced [5].

The Gensini score is a scoring system widely used to evaluate findings obtained during coronary angiography, and it objectively quantifies the severity and extent of CAD [6,7]. This system integrates the number of affected vessels with the degree of stenosis in each vessel, thereby providing a comprehensive assessment of coronary atherosclerotic burden and possessing independent prognostic value [8]. Traditional risk factors, including smoking, dyslipidemia, hypertension, diabetes, and male sex, are among the determinants of atherosclerotic burden in premature MI [9]. Lipid profile parameters are as strong as traditional risk factors in determining atherogenic lipid burden and play an important role in cardiovascular risk prediction among young patients [10]. Among lipid-derived indices, Castelli risk index 2 (CRI-2), defined as the ratio of low-density lipoprotein cholesterol (LDL-C) to high-density lipoprotein cholesterol (HDL-C), reflects the balance between atherogenic LDL-C and anti-atherogenic HDL-C and showed greater discriminatory performance than LDL-C or HDL-C alone in a retrospective angiographic study [11]. In addition, studies investigating the relationship between lymphocyte-based inflammatory indices (LBIIs) and coronary atherosclerotic burden have also been reported in the literature [12]. In a related context, composite indices incorporating the neutrophil-to-lymphocyte ratio (NLR) have been investigated as prognostic markers for all-cause mortality in patients with STEMI undergoing primary PCI [13]. However, studies specifically investigating the independent clinical, laboratory, and lipid-derived factors associated with coronary atherosclerotic burden in young patients with NSTEMI, as well as the magnitude of these associations, remain limited [14].

This study aimed to evaluate factors associated with coronary atherosclerotic burden in patients aged 45 years or younger who presented with NSTEMI. Specifically, we evaluated univariable correlations of demographic and clinical variables, laboratory parameters, and lipid and LBIIs with the Gensini score and assessed their associations after multivariable adjustment.

## 2. Materials and Methods

### 2.1. Study Participants

This single-center retrospective cross-sectional study was conducted at a tertiary hospital between January 2023 and December 2024 and is reported in accordance with the STROBE statement for observational studies. A total of 402 patients’ coronary angiography images, medical records, and laboratory findings were retrospectively evaluated. NSTEMI was defined by a rise and/or fall in cardiac troponin, with at least one value above the 99th-percentile upper reference limit, in the absence of persistent ST-segment elevation and with ischemic symptoms and/or electrocardiography (ECG) changes. Patients were eligible if they were aged ≤45 years, had troponin-positive NSTEMI, had undergone coronary angiography, and had sufficient clinical, laboratory, and angiographic data for eligibility assessment and outcome evaluation. After application of the exclusion criteria, 114 patients were excluded from the study. The reasons for exclusion were repeated coronary angiography (*n* = 30), unavailable imaging data (*n* = 9), a history of revascularization (*n* = 27; 25 percutaneous coronary interventions [PCI] and two coronary artery bypass grafting [CABG] procedures), coronary artery ectasia (*n* = 6), spontaneous coronary artery dissection (*n* = 3), slow coronary flow (*n* = 5), hemoglobin level < 10 g/dL (*n* = 12), advanced renal failure (*n* = 2), severe hypertriglyceridemia (*n* = 2), and the presence of malignancy, autoimmune disease, or active infection (*n* = 18).

### 2.2. Clinical and Laboratory Variables

The demographic characteristics of all patients and the laboratory parameters measured in blood samples collected upon admission to the coronary intensive care unit were recorded. LBIIs were calculated as follows: neutrophil-to-lymphocyte ratio (NLR) = neutrophil count/lymphocyte count; monocyte-to-lymphocyte ratio (MLR) = monocyte count/lymphocyte count; platelet-to-lymphocyte ratio (PLR) = platelet count/lymphocyte count; systemic immune-inflammation index (SII) = neutrophil count × platelet count/lymphocyte count; and systemic inflammation response index (SIRI) = neutrophil count × monocyte count/lymphocyte count. Lipid-derived indices were calculated as follows: Castelli risk index 1 (CRI-1) = total-cholesterol (C)/HDL-C; Castelli risk index 2 (CRI-2) = LDL-C/HDL-C; atherogenic index of plasma (AIP) = log10(triglycerides/HDL-C), with both values expressed in mmol/L; triglyceride–glucose index (TyG) = ln([triglycerides (mg/dL) × glucose (mg/dL)]/2); remnant cholesterol = total-C − LDL-C − HDL-C.

### 2.3. Coronary Angiography

Coronary angiograms were independently reviewed by two cardiologists who were blinded to the clinical and laboratory data, and any discrepancies were resolved by consensus. The severity and extent of CAD were assessed using the Gensini scoring system, which incorporates the degree of coronary artery stenosis and segment-specific location multipliers. The scoring criteria for coronary artery stenosis and lesion location were as follows: no angiographically detectable stenosis was assigned 0 points; stenosis of 1–25% was assigned 1 point; 26–50% stenosis, 2 points; 51–75% stenosis, 4 points; 76–90% stenosis, 8 points; 91–99% stenosis, 16 points; and complete occlusion, 32 points. Different lesion locations had different levels of importance within the coronary circulation and therefore had to be multiplied by different location coefficients. For example, lesions in the left main coronary artery were multiplied by 5. Lesions in the proximal left anterior descending artery were multiplied by 2.5, the middle segment was multiplied by 1.5, and the distal segment by 1. Lesions in the proximal left circumflex artery were multiplied by 2.5. Lesions in the middle and distal segments of the circumflex artery, the first diagonal branch, the obtuse marginal branch, the right coronary artery, and the posterior descending artery were multiplied by 1. Lesions in the second diagonal branch and the posterolateral branch were multiplied by 0.5, and so forth [15]. For multiple lesions, each lesion was scored according to its stenosis severity and segment-specific location, and the resulting scores were summed. Diffuse disease was not separately quantified beyond the lesion-based Gensini scoring rules.

### 2.4. Statistical Analysis

The distribution of continuous variables was examined both graphically and numerically. Variables with distributions approximating normality are presented as the mean ± standard deviation, skewed variables as the median (25th–75th percentile), and categorical variables as counts and percentages. The primary endpoint was the continuous Gensini score. Because of the right-skewed distribution of the score and the presence of zero values, log2(Gensini + 1) was used in the multivariable model. The model was adjusted for age, sex, diabetes mellitus, hypertension, current smoking, left ventricular ejection fraction (LVEF), estimated glomerular filtration rate (eGFR), log2-transformed NLR, and CRI-2. Heteroskedasticity-robust HC3 standard errors were used. The coefficients were back-transformed as 2^B and are reported as proportional and percentage changes in the geometric mean of Gensini + 1. Sensitivity to the outcome scale was assessed using the raw Gensini score, square-root-transformed Gensini score, and median (q = 0.50) regression. Univariable associations were examined using Spearman correlation, and the Benjamini–Hochberg false discovery rate (FDR) correction was applied in the multiple-correlation screening. Model performance was evaluated using 10-fold cross-validation repeated 20 times (200 fold-specific model fits in total), and multicollinearity was assessed using the variance inflation factor (VIF). Model diagnostics included the Breusch–Pagan test for heteroscedasticity, the Jarque–Bera test for residual normality, and the maximum Cook’s distance for influential observations. Predictive performance was summarized using the root mean squared error (RMSE) and mean absolute error (MAE) on the log2(Gensini + 1) scale and, after back-transformation, on the Gensini scale. Calibration was assessed using a calibration slope based on observed and model-predicted outcomes. The primary analysis used 281 of 288 patients (97.6%) with complete covariate data. Because HbA1c was available for only 62 of 288 patients, it was not included in the primary adjusted model. LVEF was missing in four patients and CRI-2 in three, with no overlap. The missing-data mechanism could not be established from the available records; a missing-at-random assumption conditional on the observed model variables was used for sensitivity analysis but could not be verified. Twenty multiply imputed datasets were generated using chained Bayesian ridge models with posterior sampling, and estimates were pooled using Rubin’s rules. Among the lipid-derived indices, the CRI-2 was carried into the multivariable model because these indices were strongly intercorrelated (Spearman ρ up to 1.00); this selection followed inspection of the univariable results and is therefore exploratory rather than prespecified (Supplementary Table S2). The continuous-outcome strategy and lipid-index selection were not prospectively prespecified. FDR correction was applied separately to the 52 univariable correlations, five nonlinear tests, and eight interaction tests; the primary multivariable and sensitivity analyses were reported with nominal *p* values and 95% confidence intervals. The reference category for current smoking comprised 60 never-smokers and 9 former smokers. Cross-validated performance metrics were summarized as the mean across the 20 repetitions, together with the 2.5th–97.5th percentile interval of repeat-level estimates.

All tests were two-sided, and a *p*-value of <0.05 was considered the threshold for statistical significance.

## 3. Results

The mean Gensini score was 37.9 ± 32.4, the median was 32.0 (IQR 9.4–58.1), and the range was 0–143 (Table 1). Twenty-six patients (9.0%) had a Gensini score of zero, that is, no angiographically detectable coronary stenosis. A zero Gensini score identifies an angiographically non-obstructive subgroup but does not establish MINOCA. MINOCA was not formally adjudicated, and alternative diagnoses, including myocarditis, Takotsubo syndrome, and coronary microvascular disease, were not systematically excluded. These patients were retained in the primary analysis because the outcome was defined on a continuous burden scale on which the absence of disease is a meaningful value. Of the 262 patients with a Gensini score greater than zero, 256 had complete data for all model covariates and were included in the sensitivity analysis. The primary multivariable analysis included 281 patients with complete covariate data; results from the multiple-imputation sensitivity analysis are reported in Supplementary Table S3.

In this complete-case sensitivity analysis (*n* = 256), the direction of every association was preserved, although the magnitudes were attenuated: current smoking, +42.4% (*p* = 0.059); male sex, +56.6% (*p* = 0.038); each 5-year increment in age, +16.7% (*p* = 0.079); each 5-percentage-point increase in LVEF, −13.0% (*p* = 0.014); and each 1-unit increment in CRI-2, +15.2% (*p* = 0.042). The attenuation of the smoking and age estimates suggests that the primary associations may partly reflect the contrast between the presence and absence of angiographic disease rather than a gradient of burden among patients with detectable disease; however, this pattern does not constitute a formal test of heterogeneity (Supplementary Table S4).

In the univariable analysis, the Gensini score was inversely associated with LVEF (Spearman’s ρ = −0.319; FDR q < 0.001) and positively associated with CRI-1 (ρ = 0.315; FDR q < 0.001) and CRI-2 (ρ = 0.306; FDR q < 0.001). No significant univariable association was identified between the Gensini score and NLR (Table 2; Supplementary Table S1).

In the adjusted log2 (Gensini + 1) model, each 5-year increase in age was associated with a 33.9% higher geometric mean of Gensini + 1 (95% CI, 7.6–66.6%; *p* = 0.009). Male sex was associated with a 90.1% higher value (95% CI, 14.5–215.7%; *p* = 0.013), while current smokers had a 114.3% higher geometric mean of Gensini + 1 than participants who were not currently smoking (95% CI, 39.7–228.5%; *p* < 0.001). Each 5-percentage-point increase in LVEF was associated with an 18.4% lower value (95% CI, 7.8–27.8% decrease; *p* = 0.001), whereas each 1-unit increase in the CRI-2 was associated with a 23.3% higher value (95% CI, 5.4–44.2%; *p* = 0.009). Diabetes mellitus did not meet the prespecified significance threshold (*p* = 0.054); hypertension, eGFR, and NLR were not statistically significant after multivariable adjustment. Corresponding percentage differences and 95% confidence intervals are illustrated in Figure 1 and presented in Table 3. Because CRI-2 was selected after inspection of the univariable results, its multivariable association is exploratory rather than confirmatory. None of the five nonlinear tests or eight sex-by-covariate interactions met the FDR threshold; although the sex-by-age interaction had a nominal *p* value of 0.019, its FDR-adjusted q value was 0.149 (Supplementary Tables S5 and S6).

In the multiple-imputation sensitivity analysis, the pooled estimates were generally consistent with the complete-case results, including age (+33.2%), male sex (+101.4%), current smoking (+114.3%), LVEF (−18.4%), and CRI-2 (+22.9%). Diabetes mellitus became nominally significant after imputation (+46.2%, 95% CI, 3.7–106.3%; *p* = 0.030), compared with *p* = 0.054 in the complete-case analysis. Complete pooled estimates are provided in Supplementary Table S3. Current smoking was associated with an 11.4-point higher Gensini score in the raw Gensini model (95% CI, 2.6–20.2; *p* = 0.011) and a 14.5-point higher score in the median regression model (95% CI, 4.1–24.9; *p* = 0.006). The direction and statistical significance of the associations for age, male sex, current smoking, and LVEF were maintained across all outcome scales (Table 4). In the sensitivity analyses, the CRI-2 association was statistically significant in the log2-transformed and square-root-transformed models but not in the raw Gensini and median regression models (*p* = 0.065 and *p* = 0.061, respectively).

The primary model had an apparent R^2^ of 0.278 and an adjusted R^2^ of 0.254; the model performance and diagnostic summary are presented in Table 5. Across 20 repetitions of 10-fold cross-validation, the mean out-of-fold R^2^ was 0.208 (2.5th–97.5th percentile, 0.190–0.222). The highest VIF was 1.18, indicating no substantial multicollinearity. An additional 2000-resample fixed-model bootstrap estimated a mean R^2^ optimism of 0.060, yielding an optimism-corrected R^2^ of 0.218 (Supplementary Table S8). These validation procedures were conditional on the selected covariates and did not correct optimism arising from the data-driven CRI-2 selection. The influential-observation sensitivity analysis is reported in Supplementary Table S7.

## 4. Discussion

In this study, 288 patients aged 45 years or younger who presented with NSTEMI were evaluated to identify factors associated with coronary atherosclerotic burden. In the multivariable model, smoking showed the strongest positive association, followed by male sex, age, and CRI-2, whereas LVEF showed an inverse association. None of the five LBIIs showed a statistically significant association with the continuous Gensini score after Benjamini–Hochberg FDR correction in univariable screening. The main findings were generally consistent across sensitivity analyses using raw and square-root-transformed Gensini models and median regression; smoking, male sex, age, and LVEF remained consistently associated with burden, whereas CRI-2 was statistically significant in the log2-transformed and square-root-transformed models but not in the raw Gensini and median regression models (*p* = 0.065 and *p* = 0.061, respectively). The loss of nominal significance for smoking and age after excluding patients with Gensini scores of zero suggests that these associations may partly reflect the contrast between the presence and absence of angiographic disease.

Smoking showed the strongest association with coronary atherosclerotic burden and was associated with a 114.3% higher geometric mean of Gensini + 1 in the multivariable model. This association may reflect the multifaceted adverse cardiovascular effects of smoking. Smoking induces endothelial dysfunction, thereby reducing nitric oxide bioavailability and increasing the release of superoxide anion and endothelin [16]. Moreover, through endothelial dysfunction and oxidative stress, smoking increases adhesion molecule expression, thereby enhancing platelet and macrophage adhesion. By facilitating transendothelial migration and the uptake of oxidized lipoproteins, it contributes to early atherogenesis and foam-cell formation [17]. Current smoking status was prevalent in our cohort (76%). Although the cross-sectional design precludes causal inference, this association is consistent with the known adverse effects of smoking in early coronary artery disease. Consistent with these findings, smoking-induced vascular endothelial dysfunction is regarded as an early key event in the initiation and progression of smoking-related atherosclerosis [18]. Therefore, smoking cessation interventions should be considered a fundamental component of secondary prevention in young patients with NSTEMI [19].

In the multivariable model, male patients had a 90.1% higher coronary atherosclerotic burden. This sex difference may reflect the contrast between the atherogenic effects of testosterone and the protective vascular effects of estrogen [20]. The higher prevalence of behavioral risk factors such as smoking and alcohol consumption among male patients may also contribute to this difference [21]. Each 5-year increase in age was associated with a 33.9% higher coronary atherosclerotic burden. Although this finding is consistent with greater atherosclerotic burden at older ages within a young cohort, the cross-sectional design does not establish the rate of atherosclerosis progression or its underlying mechanisms [22]. Taken together, these findings underscore the importance of traditional risk factors and early risk stratification in young patients with NSTEMI.

LVEF was inversely associated with atherosclerotic burden; each 5-percentage-point increase in LVEF corresponded to an 18.4% lower geometric mean of Gensini + 1. However, this association should not be interpreted as evidence of a protective effect of LVEF. LVEF may instead be a consequence of atherosclerotic burden rather than an upstream cause, as greater ischemic involvement may impair myocardial function. Moreover, LVEF was measured during the index hospitalization, when it may have been transiently depressed by myocardial stunning. Accordingly, we interpret the LVEF–Gensini relationship as an association and a potential downstream marker of atherosclerotic burden, rather than as an independent determinant [23,24].

In the exploratory multivariable model, each 1-unit increase in CRI-2 was associated with a 23.3% higher geometric mean of Gensini + 1 (95% CI, 5.4–44.2%; *p* = 0.009). CRI-2 is an indicator that reflects the balance between atherogenic LDL-C and anti-atherogenic HDL-C and is associated with the severity of coronary atherosclerotic burden [11]. Although the other lipid indices were also associated with atherosclerotic burden in univariable analyses, only CRI-2 was included in the multivariable model because of the strong correlations among these indices. This data-informed selection should not be interpreted as evidence that CRI-2 is superior to its component measures or other lipid indices, and its nominal association remains vulnerable to post-selection optimism. In the sensitivity analyses, the CRI-2 association was statistically significant in the log2-transformed and square-root-transformed models but not in the raw Gensini and median regression models (*p* = 0.065 and *p* = 0.061, respectively). This finding is consistent with previous observational studies linking higher LDL-C/HDL-C ratios to coronary atherosclerotic severity, acute MI risk, and adverse cardiovascular outcomes [11,25].

Although LBIIs (NLR, MLR, PLR, SII, and SIRI) have shown significant correlations with the Gensini or SYNTAX scores in general CAD and acute coronary syndrome populations, these indices did not show a significant univariable association with coronary atherosclerotic burden in NSTEMI patients aged 45 years or younger in our study [12,26,27,28]. One possible explanation is that the Gensini score reflects the extent of coronary lesions, whereas LBIIs measured during the acute phase of NSTEMI may reflect the acute inflammatory response rather than chronic vascular inflammation. Because these measures represent different biological states, their correlation may be weak.

The principal message of this study is that, among patients aged 45 years or younger presenting with NSTEMI, smoking, male sex, older age, and CRI-2 were associated with greater coronary atherosclerotic burden in the primary multivariable model. However, the CRI-2 association was not consistent across all sensitivity models, whereas none of the LBIIs examined showed a significant univariable association after FDR correction. By evaluating clinical variables, lipid-derived indices, and LBIIs within a common analytical framework, this study provides a focused assessment of angiographic burden in young patients with NSTEMI. These findings support smoking cessation and guideline-concordant lipid management as key modifiable priorities for secondary prevention in this population.

### Limitations

Several limitations should be acknowledged. First, the retrospective, single-center design precludes causal inference and may introduce selection, information, and residual confounding biases, thereby limiting generalizability. The exclusion of patients with prior revascularization or coronary ectasia may have further contributed to selection bias. Formal MINOCA adjudication was not performed, and myocarditis, Takotsubo syndrome, and coronary microvascular disease were not systematically excluded. Although the assessors were blinded and discrepancies were resolved by consensus, interobserver agreement was not formally quantified using an intraclass correlation coefficient. Second, women comprised only 16.7% of the cohort; therefore, the estimate for male sex was imprecise and sex-stratified analyses were not feasible. Third, the absence of long-term follow-up precluded assessment of the prognostic significance of angiographic burden. Fourth, LBIIs and lipid measurements were obtained from blood samples collected upon admission to the coronary intensive care unit; however, the interval from symptom onset to sampling, fasting status, and whether sampling preceded treatment initiation were not systematically recorded. These factors limit the interpretation of the laboratory measurements and may have introduced measurement variability. Fifth, because of the retrospective design and the lack of standardized recording, BMI, family history of premature coronary disease, pack-years, alcohol use, pre-admission lipid-lowering therapy, and in-hospital treatment were not systematically available. In addition, infarct territory, culprit vessel, and a standardized diseased-vessel count were not collected as part of the present analysis. Lipoprotein(a) and apolipoprotein B were unavailable, and HbA1c was missing in 226 of 288 patients (78.5%); these omissions limited adjustment for potentially relevant clinical factors and may have contributed to residual confounding and diabetes misclassification. Sixth, CRI-2 selection was data-informed: its nominal inference did not account for post-selection optimism, and its association was not consistent across all outcome scales; therefore, it requires confirmation. Finally, the model explained a modest proportion of the variance and was not intended for individual-level prediction. Prospective, multicenter studies with external validation, standardized measurements, adequate representation of women, and long-term follow-up are needed to confirm these findings.

## 5. Conclusions

In this single-center retrospective study of patients aged 45 years or younger presenting with NSTEMI, current smoking, male sex, older age, and higher CRI-2 values were associated with greater coronary atherosclerotic burden. However, because CRI-2 was selected after inspection of the univariable results, this finding should be considered exploratory. None of the five LBIIs showed a statistically significant association with the continuous Gensini score after Benjamini–Hochberg FDR correction in univariable screening. These findings reinforce the importance of smoking cessation and guideline-directed lipid management in secondary prevention, although this cross-sectional study did not evaluate treatment effects.

## Figures and Tables

**Figure 1:**
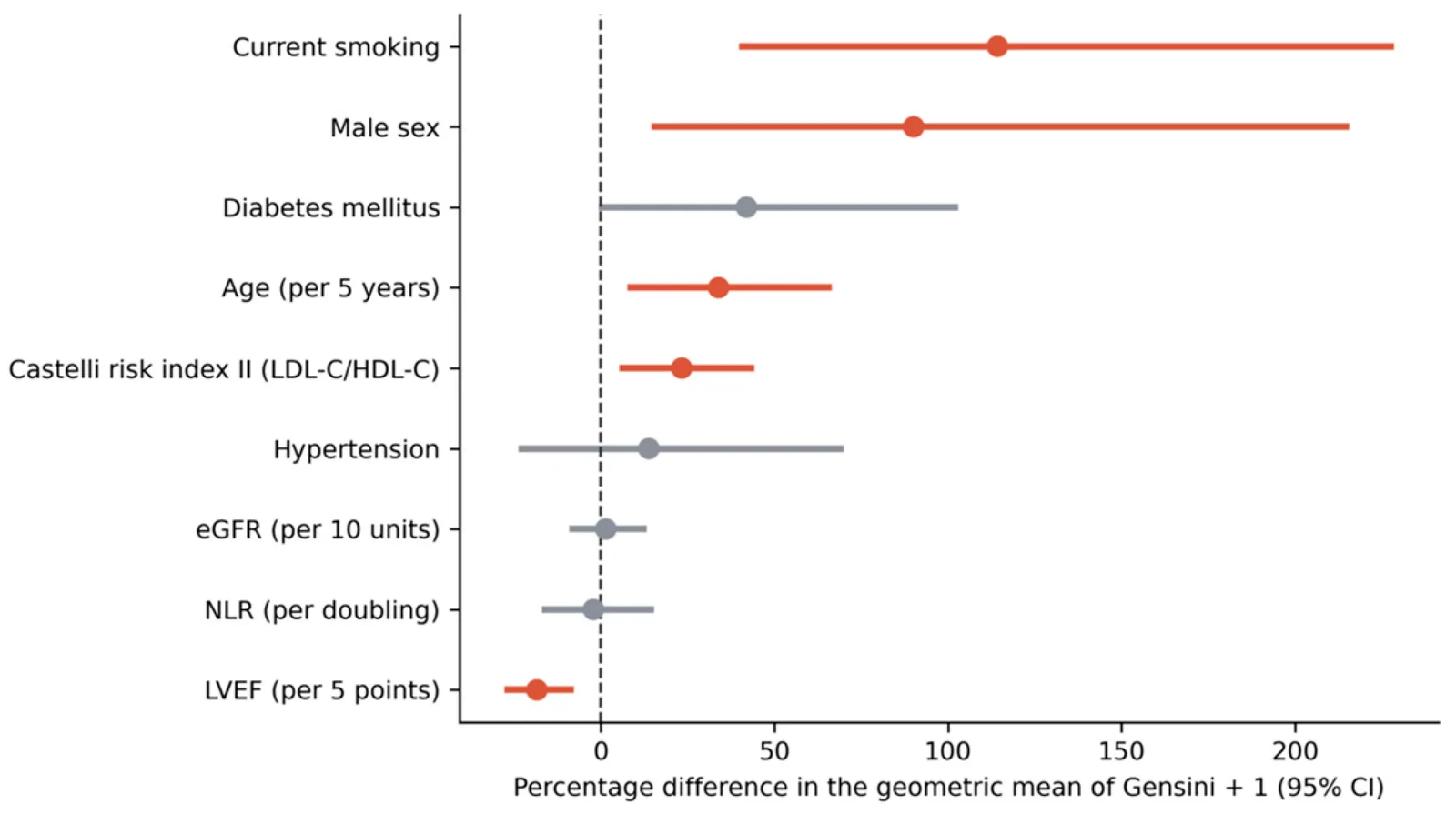
Forest plot of the primary multivariable model. Points show percentage differences in the geometric mean of Gensini + 1; horizontal lines indicate 95% CIs.

**Table 1:** Demographic, clinical, and laboratory characteristics of the study cohort.

Variable	*N*	Presentation	Value	Missing *n*
Male sex	288	*n* (%)	240 (83.3)	0
Age, years	288	Mean ± SD	40.7 ± 4.1	0
Diabetes mellitus	288	*n* (%)	62 (21.5)	0
Hypertension	288	*n* (%)	61 (21.2)	0
Current smoking	288	*n* (%)	219 (76.0)	0
Former smoking	288	*n* (%)	9 (3.1)	0
LVEF, %	284	Mean ± SD	57.0 ± 7.7	4
Gensini score	288	Median (IQR)	32.0 (9.4–58.1)	0
hs-Troponin T, ng/L	286	Median (IQR)	205.0 (77.5–737.2)	2
Glucose, mg/dL	285	Median (IQR)	101.0 (87.0–121.0)	3
Creatinine, mg/dL	288	Median (IQR)	0.9 (0.8–1.0)	0
eGFR, mL/min/1.73 m^2^	288	Mean ± SD	96.7 ± 14.1	0
Total-C, mg/dL	284	Mean ± SD	219.3 ± 52.3	4
HDL-C, mg/dL	285	Mean ± SD	43.5 ± 10.0	3
LDL-C, mg/dL	285	Mean ± SD	139.2 ± 43.9	3
Triglycerides, mg/dL	285	Median (IQR)	165.0 (106.0–268.0)	3
White blood cell count, 10^3^/µL	288	Median (IQR)	10.7 (8.8–12.9)	0
Hemoglobin, g/dL	288	Mean ± SD	14.6 ± 1.6	0
Platelet count, 10^3^/µL	288	Median (IQR)	261.5 (222.8–310.0)	0
NLR	288	Median (IQR)	2.6 (1.8–4.0)	0
MLR	288	Median (IQR)	0.3 (0.2–0.4)	0
PLR	288	Median (IQR)	99.0 (78.7–135.2)	0
SII	288	Median (IQR)	664.8 (444.5–1171.4)	0
SIRI	288	Median (IQR)	2.0 (1.3–3.2)	0
CRI-1	284	Median (IQR)	5.0 (4.3–5.9)	4
CRI-2	285	Median (IQR)	3.2 (2.5–3.9)	3
Triglyceride/HDL-C	285	Median (IQR)	4.1 (2.4–6.4)	3
AIP	285	Median (IQR)	0.2 (0.0–0.4)	3
TyG	282	Median (IQR)	9.1 (8.5–9.7)	6
Remnant-C, mg/dL	284	Median (IQR)	32.0 (21.0–48.0)	4
HbA1c, %	62	Median (IQR)	6.8 (5.9–9.4)	226

Values are presented as mean ± standard deviation, median (25th–75th percentile), or *n* (%). The Gensini score was analyzed as a continuous outcome in the primary analysis. AIP, atherogenic index of plasma; CRI-1, Castelli risk index 1; CRI-2, Castelli risk index 2; eGFR, estimated glomerular filtration rate; HDL-C, high-density lipoprotein cholesterol; hs-Troponin T, high-sensitivity cardiac troponin T; IQR, interquartile range; LDL-C, low-density lipoprotein cholesterol; LVEF, left ventricular ejection fraction; MLR, monocyte-to-lymphocyte ratio; NLR, neutrophil-to-lymphocyte ratio; PLR, platelet-to-lymphocyte ratio; Remnant-C, remnant cholesterol; SII, systemic immune-inflammation index; SIRI, systemic inflammation response index; Total-C, total cholesterol; TyG, triglyceride–glucose index; HbA1c, glycated hemoglobin.

**Table 2:** Spearman correlations of selected variables with the continuous Gensini score.

Variable	*N*	Spearman ρ	*p*	FDR q	Direction and Magnitude
Age, years	288	0.159	0.007	0.019	weak, positive
LVEF, %	284	−0.319	<0.001	<0.001	moderate, negative
hs-Troponin T, ng/L	286	0.151	0.010	0.027	weak, positive
Total-C, mg/dL	284	0.218	<0.001	0.001	weak, positive
HDL-C, mg/dL	285	−0.093	0.116	0.208	negligible, negative
LDL-C, mg/dL	285	0.233	<0.001	<0.001	weak, positive
Triglycerides, mg/dL	285	0.214	<0.001	0.001	weak, positive
CRI-1	284	0.315	<0.001	<0.001	moderate, positive
CRI-2	285	0.306	<0.001	<0.001	moderate, positive
Triglyceride/HDL-C	285	0.215	<0.001	0.001	weak, positive
AIP	285	0.215	<0.001	0.001	weak, positive
TyG	282	0.227	<0.001	<0.001	weak, positive
Remnant-C, mg/dL	284	0.179	0.002	0.008	weak, positive
NLR	288	0.023	0.701	0.775	negligible, positive
MLR	288	0.088	0.135	0.226	negligible, positive
PLR	288	−0.067	0.255	0.390	negligible, negative
SII	288	0.019	0.750	0.796	negligible, positive
SIRI	288	0.118	0.045	0.086	weak, positive

Correlations are univariable and unadjusted for potential confounders. FDR q values were obtained by applying the Benjamini–Hochberg procedure to all 52 correlations. AIP, atherogenic index of plasma; CRI-1, Castelli risk index 1; CRI-2, Castelli risk index 2; FDR, false discovery rate; HDL-C, high-density lipoprotein cholesterol; hs-Troponin T, high-sensitivity cardiac troponin T; LDL-C, low-density lipoprotein cholesterol; LVEF, left ventricular ejection fraction; MLR, monocyte-to-lymphocyte ratio; NLR, neutrophil-to-lymphocyte ratio; PLR, platelet-to-lymphocyte ratio; SII, systemic immune-inflammation index; SIRI, systemic inflammation response index; Total-C, total cholesterol; TyG, triglyceride–glucose index; ρ, Spearman correlation coefficient.

**Table 3:** Primary multivariable model for log2(Gensini + 1).

Predictor	B	Robust SE	*p*	2^B	95% CI	Percentage Difference (95% CI)	VIF
Age (per 5-year increase)	0.421	0.161	0.009	1.34	1.08–1.67	33.9% (7.6% to 66.6%)	1.14
Male sex (vs. female)	0.927	0.373	0.013	1.90	1.14–3.16	90.1% (14.5% to 215.7%)	1.16
Diabetes mellitus (vs. absent)	0.506	0.263	0.054	1.42	0.99–2.03	42.0% (−0.7% to 102.9%)	1.11
Hypertension (vs. absent)	0.187	0.295	0.527	1.14	0.76–1.70	13.8% (−23.8% to 70.0%)	1.12
Current smoking (vs. not currently smoking)	1.099	0.315	<0.001	2.14	1.40–3.29	114.3% (39.7% to 228.5%)	1.18
LVEF (per 5-percentage-point increase)	−0.294	0.090	0.001	0.82	0.72–0.92	−18.4% (−27.8% to −7.8%)	1.08
eGFR (per 10-unit increase)	0.020	0.081	0.804	1.01	0.91–1.13	1.4% (−9.2% to 13.2%)	1.05
NLR (per doubling)	−0.031	0.121	0.796	0.98	0.83–1.15	−2.1% (−17.0% to 15.3%)	1.09
CRI-2 (per 1-unit increase)	0.302	0.116	0.009	1.23	1.05–1.44	23.3% (5.4% to 44.2%)	1.16

The dependent variable was log2(Gensini + 1). Here, 2^B is the geometric mean ratio for Gensini + 1 and is not an odds ratio. Current smoking is a binary status variable; patients who were not current smokers constituted the reference group. Model *n* = 281. B, regression coefficient; CI, confidence interval; CRI-2, Castelli risk index 2; eGFR, estimated glomerular filtration rate; LVEF, left ventricular ejection fraction; NLR, neutrophil-to-lymphocyte ratio; SE, standard error; VIF, variance inflation factor.

**Table 4:** Outcome-scale sensitivity analyses: raw, square-root, and median Gensini models.

Predictor	Raw Gensini B (95% CI)	*p*	Square-Root Gensini B (95% CI)	*p*	Median Gensini B (95% CI)	*p*
Age (per 5-year increase)	5.1 (0.8–9.5)	0.021	0.59 (0.14–1.03)	0.010	6.4 (1.0–11.7)	0.019
Male sex (vs. female)	12.8 (3.7–21.9)	0.006	1.35 (0.36–2.33)	0.008	16.3 (4.6–28.1)	0.007
Diabetes mellitus (vs. absent)	5.5 (−4.4–15.5)	0.275	0.65 (−0.20–1.50)	0.132	2.3 (−8.1–12.8)	0.662
Hypertension (vs. absent)	1.8 (−8.5–12.0)	0.732	0.24 (−0.68–1.15)	0.614	1.8 (−8.7–12.4)	0.733
Current smoking (vs. not currently smoking)	11.4 (2.6–20.2)	0.011	1.43 (0.57–2.30)	0.001	14.5 (4.1–24.9)	0.006
LVEF (per 5-percentage-point increase)	−5.9 (−9.1–−2.6)	<0.001	−0.51 (−0.80–−0.23)	<0.001	−6.5 (−9.2–−3.8)	<0.001
eGFR (per 10-unit increase)	0.1 (−2.7–2.8)	0.963	0.03 (−0.22–0.27)	0.832	2.0 (−1.1–5.0)	0.202
NLR (per doubling)	−1.1 (−5.1–2.9)	0.591	−0.06 (−0.43–0.30)	0.732	−1.0 (−5.8–3.7)	0.674
CRI-2 (per 1-unit increase)	3.6 (−0.2–7.4)	0.065	0.43 (0.08–0.77)	0.017	4.1 (−0.2–8.5)	0.061

Raw-score and square-root models used HC3-robust linear regression; the median model used q = 0.50 quantile regression. Coefficients in the raw-score and median models represent Gensini-point differences. Median regression does not dichotomize patients at the sample median. B, regression coefficient; CI, confidence interval; CRI-2, Castelli risk index 2; eGFR, estimated glomerular filtration rate; LVEF, left ventricular ejection fraction; NLR, neutrophil-to-lymphocyte ratio.

**Table 5:** Performance and diagnostic summary of the primary model.

Domain	Metric	Value	Interpretation
Model fit	Apparent R^2^	0.278	Variance explained in the development data
Model fit	Adjusted R^2^	0.254	Adjusted for the number of predictors
Cross-validation	Repeated 10-fold R^2^	0.208 (0.190–0.222)	Out-of-fold explained variance
Cross-validation	RMSE (log2 scale)	1.774 (1.758–1.794)	Lower values indicate better performance
Cross-validation	MAE (log2 scale)	1.411 (1.400–1.431)	Lower values indicate better performance
Cross-validation	RMSE (Gensini scale)	35.3 (34.6–36.1) points	Approximate prediction error
Cross-validation	MAE (Gensini scale)	25.3 (25.0–25.6) points	Approximate absolute error
Calibration	Slope	0.861 (0.828–0.896)	A value near 1 is desirable
Diagnostics	Breusch–Pagan *p*	<0.001	Heteroscedasticity present; HC3 used
Diagnostics	Jarque–Bera *p*	<0.001	Residuals were not fully normal
Diagnostics	Maximum Cook’s distance	0.101	Below 1
Diagnostics	Maximum VIF	1.18	No relevant multicollinearity

Cross-validation estimates are means from 20 repetitions of shuffled 10-fold cross-validation (200 fold specific model fits); values in parentheses represent the 2.5th–97.5th percentile interval across repetitions. MAE, mean absolute error; RMSE, root mean squared error; R^2^, coefficient of determination; VIF, variance inflation factor.

## Data Availability

The data presented in this study are available on request from the corresponding author.

## Supplementary Materials

The following supporting information can be downloaded at https://www.mdpi.com/article/10.3390/jcm15197442/s1. Table S1: All 52 Spearman correlations with the continuous Gensini score; Table S2: Alternative lipid variables substituted into the primary model; Table S3: Multiple imputation sensitivity analysis; Table S4: Sensitivity analysis excluding patients with a Gensini score of zero; Table S5: Exploratory tests for nonlinearity; Table S6: Exploratory sex-by-covariate interactions; Table S7: Influential-observation sensitivity analysis; Table S8: Fixed-model internal validation.
