# Supplementary material for: The Correlation of Clinical, Lipid- and Inflammation-Based Indices with Gensini Score in Young NSTEMI Patients
Source: J Clin Med. 2026 Sep 24;15(19):7442. doi: 10.3390/jcm15197442 (PMC13666487; doi:10.3390/jcm15197442)
Supplement: Supplementary file 1 [file jcm-15-07442-s001.zip › jcm-4557110-supplementary.pdf]

SUPPLEMENTARY MATERIAL

Supplementary Table S1: All 52 Spearman correlations with the continuous Gensini score.

	Variable	N	Spearman ρ	p	FDR q
1	LVEF	284	-0.319	<0.001	<0.001
2	CRI-1	284	0.315	<0.001	<0.001
3	Atherogenic coefficient (AC)	284	0.315	<0.001	<0.001
4	CRI-2	285	0.306	<0.001	<0.001
5	TG×AC (study-specific composite index)	284	0.263	<0.001	<0.001
6	Non-HDL-C	284	0.246	<0.001	<0.001
7	LDL-C	285	0.233	<0.001	<0.001
8	TyG	282	0.227	<0.001	<0.001
9	Total-C	284	0.218	<0.001	0.001
10	Triglyceride/HDL-C	285	0.215	<0.001	0.001
11	AIP	285	0.215	<0.001	0.001
12	VLDL-C	285	0.214	<0.001	0.001
13	Triglycerides	285	0.214	<0.001	0.001
14	Hemoglobin	288	0.201	<0.001	0.002
15	Hematocrit	288	0.179	0.002	0.008
16	Remnant-C	284	0.179	0.002	0.008
17	MHR	285	0.171	0.004	0.011
18	Monocyte count	288	0.165	0.005	0.015
19	Age	288	0.159	0.007	0.019
20	hs-Troponin T	286	0.151	0.010	0.027
21	Basophil count	288	0.144	0.014	0.035
22	NHR	285	0.139	0.019	0.044
23	Creatinine	288	0.137	0.020	0.045

	Variable	N	Spearman ρ	p	FDR q
24	White blood cell count	288	0.135	0.022	0.048
25	ALT	282	0.129	0.030	0.062
26	IG	285	0.121	0.041	0.083
27	SIRI	288	0.118	0.045	0.086
28	Neutrophil count	288	0.114	0.054	0.100
29	HDL-C	285	-0.093	0.116	0.208
30	SIIRI	288	0.092	0.120	0.208
31	MLR	288	0.088	0.135	0.226
32	Sodium	288	-0.081	0.172	0.280
33	Glucose	285	0.072	0.225	0.355
34	PLR	288	-0.067	0.255	0.390
35	Potassium	288	-0.065	0.273	0.398
36	Lymphocyte count	288	0.064	0.276	0.398
37	LDH	259	0.066	0.293	0.411
38	RDW-CV	288	-0.051	0.387	0.530
39	eGFR	288	-0.043	0.469	0.594
40	HbA1c	62	0.094	0.470	0.594
41	Albumin	237	0.047	0.470	0.594
42	Uric acid	242	0.046	0.480	0.594
43	AST	270	-0.041	0.499	0.604
44	RDW-SD	288	0.034	0.564	0.666
45	Plateletcrit	285	0.032	0.596	0.689
46	BUN	288	-0.023	0.695	0.775
47	NLR	288	0.023	0.701	0.775
48	Total bilirubin	257	0.022	0.727	0.787

	Variable	N	Spearman ϱ	p	FDR q
49	SII	288	0.019	0.750	0.796
50	Magnesium	264	-0.012	0.842	0.876
51	Platelet count	288	0.007	0.902	0.920
52	Eosinophil count	288	-0.004	0.940	0.940

Values are ordered by nominal p value. N, number of non-missing observations; AC, atherogenic coefficient; AIP, atherogenic index of plasma; ALT, alanine aminotransferase; AST, aspartate aminotransferase; BUN, blood urea nitrogen; CRI-1, Castelli risk index 1; CRI-2, Castelli risk index 2; eGFR, estimated glomerular filtration rate; FDR, false discovery rate; HbA1c, glycated hemoglobin; HDL-C, high-density lipoprotein cholesterol; hs-Troponin T, high-sensitivity cardiac troponin T; IG, immature granulocyte count; LDH, lactate dehydrogenase; LVEF, left ventricular ejection fraction; MHR, monocyte-to-high-density lipoprotein cholesterol ratio; MLR, monocyte-to-lymphocyte ratio; NHR, neutrophil-to-high-density lipoprotein cholesterol ratio; NLR, neutrophil-to-lymphocyte ratio; PLR, platelet-to-lymphocyte ratio; RDW-CV, red cell distribution width–coefficient of variation; RDW-SD, red cell distribution width–standard deviation; SII, systemic immune-inflammation index; SIRI, systemic inflammation response index; SIIRI, systemic immune-inflammatory response index; Total-C, total cholesterol; TyG, triglyceride–glucose index; VLDL-C, very-low-density lipoprotein cholesterol; ϱ, Spearman correlation coefficient. FDR q values were calculated using the Benjamini–Hochberg procedure across all 52 tests. AC was calculated as [(Total-C − HDL-C) / HDL-C]. TG×AC was calculated as [TG × [(Total-C − HDL-C) / HDL-C]]. SIIRI was calculated as (neutrophil count × monocyte count × platelet count) / lymphocyte count.

Supplementary Table S2: Alternative lipid variables substituted into the primary model.

Lipid variable substituted	N	p	Model R^2	ΔR^2 (bootstrap 95% CI)
Total-C	280	<0.001	0.302	0.044 (0.012–0.090)
Non-HDL-C	280	<0.001	0.302	0.044 (0.010–0.093)
LDL-C	281	<0.001	0.292	0.034 (0.007–0.071)
CRI-1	280	0.004	0.281	0.023 (0.002–0.060)
CRI-2	281	0.009	0.278	0.020 (0.002–0.059)
TyG	278	0.034	0.271	0.014 (0.000–0.050)
Remnant-C	280	0.078	0.269	0.012 (0.000–0.044)
AIP	281	0.098	0.266	0.008 (0.000–0.034)
Triglyceride/HDL-C	281	0.112	0.264	0.006 (0.000–0.027)

Each lipid variable was substituted one at a time for CRI-2 in the otherwise unchanged multivariable model; the CRI-2 row represents the primary model. ΔR^2 denotes the increase in R^2 relative to the same model without a lipid term; 95% confidence intervals were obtained from 2000 bootstrap resamples. Confidence intervals overlapped substantially; this analysis does not establish that any one index is superior and does not support the claim that the LDL-C/HDL-C ratio outperforms its components. AIP, atherogenic index of plasma; CRI-2, Castelli risk index 2; HDL-C, high-density lipoprotein cholesterol; LDL-C, low-density lipoprotein cholesterol; R^2, coefficient of determination; Total-C, total cholesterol; TyG, triglyceride–glucose index.

Supplementary Table S3: Multiple imputation sensitivity analysis.

Predictor	Multiple imputation, % (95% CI)	p	Complete-case analysis, %	p
Age (per 5 years)	33.2 (7.5 to 65.2)	0.009	33.9	0.009
Male sex	101.4 (21.8 to 232.9)	0.006	90.1	0.013
Diabetes mellitus	46.2 (3.7 to 106.3)	0.030	42.0	0.054
Hypertension	16.5 (-21.0 to 71.7)	0.441	13.8	0.527
Current smoking	114.3 (40.8 to 226.2)	<0.001	114.3	<0.001
LVEF (per 5 points)	-18.4 (-27.7 to -7.9)	0.001	-18.4	0.001
eGFR (per 10 units)	2.5 (-8.0 to 14.1)	0.658	1.4	0.804
NLR (per doubling)	-3.7 (-17.7 to 12.6)	0.633	-2.1	0.796
CRI-2	22.9 (5.3 to 43.5)	0.009	23.3	0.009

Twenty imputed datasets were generated using chained equations, and estimates were pooled using Rubin's rules. Percentages represent back-transformed percentage differences in the geometric mean of Gensini + 1 from the log2(Gensini + 1) model. Results were directionally consistent with the complete-case analysis, although diabetes mellitus reached nominal statistical significance after multiple imputation. CI, confidence interval; LVEF, left ventricular ejection fraction; eGFR, estimated glomerular filtration rate; NLR, neutrophil-to-lymphocyte ratio; CRI-2, Castelli risk index 2.

Supplementary Table S4: Sensitivity analysis excluding patients with a Gensini score of zero.

Predictor	Gensini >0 difference % 95% CI	Nominal p	Full cohort difference %
Age per 5 years	16.7 (-1.8 to 38.6)	0.079	33.9
Male sex	56.6 (2.5 to 139.3)	0.038	90.1
Diabetes mellitus	14.4 (-16.3 to 56.3)	0.399	42.0
Hypertension	18.4 (-14.6 to 64.1)	0.311	13.8
Current smoking	42.4 (-1.3 to 105.6)	0.059	114.3
LVEF per 5 points	-13.0 (-22.2 to -2.8)	0.014	-18.4
eGFR per 10 units	6.9 (-2.8 to 17.5)	0.170	1.4
NLR per doubling	-2.6 (-16.4 to 13.4)	0.732	-2.1
CRI-2 per unit	15.2 (0.5 to 32.0)	0.042	23.3

N = 256 complete cases among 262 patients with Gensini >0. The model retains log2(Gensini + 1) and all primary covariates. This is not a log2(Gensini) model or a clinically adjudicated MINOCA-exclusion analysis. No formal between-subgroup difference test is implied by comparing p values.

Supplementary Table S5: Exploratory tests for nonlinearity.

Variable	HC3 Wald χ^2	df	Nominal p	FDR q
Age5	2.372	2	0.306	0.509
EF5	4.787	2	0.091	0.355

Variable	HC3 Wald χ²	df	Nominal p	FDR q
CRI-2	3.903	2	0.142	0.355
log2(NLR)	1.299	2	0.522	0.653
eGFR10	0.736	2	0.692	0.692

N = 281. Each predictor received two additional restricted cubic spline terms, with knots at the 5th, 35th, 65th, and 95th percentiles of the complete-case sample. All other model terms remained linear and unchanged. Joint HC3-robust Wald tests assessed departure from linearity, with Benjamini–Hochberg false discovery rate correction applied across the five tests. Failure to reject the null hypothesis does not prove linearity. Age5 = age/5; EF5 = LVEF/5; eGFR10 = eGFR/10; CastelliII = CRI-2.

Supplementary Table S6: Exploratory sex-by-covariate interactions.

Sex by covariate	Interaction B	HC3 SE	Nominal p	FDR q
Age5	1.057	0.449	0.019	0.149
DM	0.308	0.820	0.707	0.778
HT	-0.765	0.868	0.378	0.606
Smoke	1.010	0.767	0.188	0.376
EF5	0.426	0.312	0.172	0.376
eGFR10	-0.091	0.324	0.778	0.778
log2NLR	-0.707	0.369	0.055	0.222
CastelliII	0.097	0.305	0.751	0.778

Each interaction was fitted separately with all nine primary-model main effects retained; none was added to the primary model. The interaction coefficient represents the male-minus-female difference in the corresponding log2(Gensini + 1) slope, not the overall sex coefficient. HC3-robust standard errors were used, and Benjamini–Hochberg false discovery rate correction was applied across the eight interaction tests. There were 47 women in the complete-case sample; therefore, interaction estimates have limited precision, and no reliable sex-specific clinical conclusions should be drawn.

Supplementary Table S7: Influential-observation sensitivity analysis.

Predictor	After diagnostic omission Difference % (95% CI)	Nominal p	Primary difference %
Age per 5 years	42.3 (18.0 to 71.7)	<0.001	33.9
Male sex	135.6 (53.7 to 261.0)	<0.001	90.1
Diabetes mellitus	47.9 (8.9 to 101.0)	0.012	42.0
Hypertension	39.7 (3.8 to 87.9)	0.027	13.8
Current smoking	134.3 (63.0 to 236.8)	<0.001	114.3
LVEF per 5 points	-21.9 (-28.6 to -14.7)	<0.001	-18.4
eGFR per 10 units	2.0 (-7.2 to 12.1)	0.678	1.4
NLR per doubling	-9.9 (-21.6 to 3.5)	0.140	-2.1
CRI-2 per unit	25.4 (10.0 to 43.0)	<0.001	23.3

N = 260 after omitting 21 observations with Cook's distance >4/281 in the original fitted model. This is an exploratory sensitivity analysis, not a data-cleaning rule or a basis for deleting valid patients or selecting a more significant model. The full-sample model (n = 281) remains the primary analysis. The maximum Cook's distance was 0.101 and does not imply absence of influence. Inference is nominal.

Supplementary Table S8: Fixed-model internal validation.

Metric	Mean	2.5th percentile	97.5th percentile
Repeated CV R²	0.208	0.190	0.222
RMSE on log2 scale	1.774	1.758	1.794

Metric	Mean	2.5th percentile	97.5th percentile
MAE on log2 scale	1.411	1.400	1.431
RMSE on Gensini scale	35.268	34.621	36.079
MAE on Gensini scale	25.273	24.982	25.631
Out-of-fold calibration slope	0.861	0.828	0.896

Shuffled 10-fold cross-validation was repeated 20 times, yielding 200 fold-specific fits. Values are reported as the mean and 2.5th–97.5th percentiles across repetitions; these percentiles do not represent population confidence intervals. A 2000-resample fixed-model bootstrap estimated a mean R^2 optimism of 0.060; the apparent R^2 was 0.278 and the optimism-corrected R^2 was 0.218. Both validation procedures were conditional on the selected covariates and did not account for the historical lipid-index selection.
